# Gender analysis is not only about gender: reshaping the potato breeding priorities to increase varietal adoption in Kenya

**DOI:** 10.3389/fsoc.2024.1446973

**Published:** 2024-12-06

**Authors:** Michael Friedmann, Vivian Polar, Thiago Mendes

**Affiliations:** ^1^Independent Consultant, Santa Barbara, CA, United States; ^2^Social and Nutritional Sciences Division, International Potato Center (CIP), Lima, Peru; ^3^Genetics, Genomics and Crop Improvement Division, International Potato Center (CIP), Nairobi, Kenya

**Keywords:** potato breeding, gender-responsive breeding, social inclusion, market segments, target product profiles

## Abstract

Public breeding programs are pushing to implement demand-led breeding to increase variety adoption, while tackling multiple challenges for increased production under climate change. This has included the improvement of variety target product profiles involving multiple stakeholders. A special case involves the unexpected and rapid spread of the Shangi potato variety in Kenya. This variety was not an especially outstanding variety and the levels of its traits did not exceed the expected thresholds defined in the target product profile for table potato in East Africa. By examining the customer segments looking at gender but also social contexts of smallholder and disadvantaged farmers such as access to markets, inputs, and labor, it became apparent that ready availability of potatoes for consumption, processing, or planting was of prime importance. Given the storage and market constraints, Shangi's very short dormancy, which had been assumed to be a negative trait for farmers, women cottage processors and consumers, was actually meeting the needs for available product and planting material. Consequently, this provided these groups increased control over their productive activities. The case study presented here analyzes different components of potato variety change in Kenya. It explores the challenges and tradeoffs faced by public sector breeding programs and how gender analysis from a broader inclusion perspective can uncover the underlying causes of varietal adoption. Focusing on the Shangi potato variety, the case study reveals a series of lessons learned that have re-shaped the definition of breeding priorities.

## 1 Introduction

Potato is the second most important staple crop in Kenya after maize. There are about 800,000 producers covering 214,600 ha of production (Kaguongo et al., 2014) with an average yield of 9.8 t/ha and total production of 2.1 million tons (FAOSTAT, 2021). However, production and consumption suffer from a number of challenges including biotic and abiotic stresses and post-harvest losses due to poor storage facilities. To address the diversity of challenges, the plant breeding programs from the International Potato Center (CIP) and Kenyan partners are developing new high-yielding varieties with improved disease and virus resistance and drought and heat tolerance. In addition, the breeders strive to maintain tuber quality. Although these traits address the main production constraints, there are other factors that play into the adoption of such varieties. The rates of adoption have been much lower than hoped for (Thiele et al., 2021; Kwambai et al., 2024). Moreover, the age of varieties still in production since release in Kenya is quite high, reaching an average of over 19 years. There are mainly two factors explaining low adoption. There is a need to consider quality traits that respond to the needs and demands of stakeholders along the potato value chains (Kisakye et al., 2020; Mudege et al., 2021). In addition, there is a lack of access to clean quality seed of new improved varieties (Okello et al., 2016; Thiele et al., 2021; Kwambai et al., 2024). A study in Ethiopia showed adoption of potato varieties was dependent on the income status of the farmers, which affected their access to improved varieties and associated technologies (Yenenesh et al., 2017).

This case study analyzes different components of potato variety change in Kenya. It explores the challenges and tradeoffs faced by public sector breeding programs and how gender analysis from a broader inclusion perspective can uncover the underlying causes of varietal adoption. Focusing on the Shangi potato variety, the case study reveals a series of lessons learned that have re-shaped the definition of breeding priorities.

## 2 The context of potato breeding and variety change in Kenya

### 2.1 Variety changes in Kenya

Shangi is a farmer selection that was taken up by farmers from on-farm trials. Preliminary molecular analysis of Shangi seems to place it in the CIP genebank germplasm (Thiele et al., 2021). It has expanded rapidly across production areas since 2005. Shangi was officially released in 2015 and now occupies around 80% of the production area^1^ in Kenya. It is a high yielding, quick maturing, short dormancy, cream skinned potato, with good tuber size, good taste and quick cooking time and moderate Late Blight resistance.^2^ However, the outstanding differences between Shangi and the rest of the varieties available in the market are its very short dormancy and early maturity that make Shangi suited to year-round production. Therefore, traders in markets and processors as well can rely on a continuous supply, and farmers are able to find planting material when needed giving them greater control over their productive activities. Indeed, improved availability of produce and planting material are major causes for adoption as reviewed in Thiele et al. (2021).

A number of surveys of the potato value chain and markets have been carried out to examine varietal preferences and elucidate the related traits. A farmer survey conducted in five main potato growing counties in Kenya found issues with supply of potato tubers for processing into chips or crisps is in part due to lack of potato storage facilities (Kaguongo et al., 2014). A market survey by Manyasa (2015) of the main destination markets for fresh ware potatoes found Shangi was the most preferred variety and most of the traders surveyed (55% male, 45% female) cited availability as the most important factor for preferring Shangi. Additionally, customers desired to have affordable potatoes available throughout the year, hence availability was a major aspect of user demand.

Good storability was identified as a major trait needed by both farmers and processors, due to a lack of good storage infrastructure across the country (Okello et al., 2017) and the high cost of cold storage which is only available for large commercial farmers or large processors (Kaguongo et al., 2010; Manyasa, 2015). Indeed, a study found that farmers generally do not have good storage facilities and typically store potato seed and food in their homes/farms (Kaguongo et al., 2010; Manyasa, 2015). For potatoes to store well for a few months in between growing seasons, as well as to wait for better market prices or to provide a steady input to large processing facilities, a variety must also have a long dormancy so that it does not sprout.

In 2017 a study looked at the potential of investing in storage facilities along the value chain in Kenya (Soethoudt and Gitau, 2017). Shangi was found to be the most popular variety, as the market is mainly for direct consumption shortly after harvesting, so long post-harvest storage was barely mentioned. Traders preferred Shangi because availability was the most important factor for them. Processors had a number of quality attributes required including a thin peel to reduce waste, so Shangi with its thicker peel was less preferred. Customers were found to choose varieties based on price, size, and quality attributes, such as taste and oil consumption when frying. In terms of storage of potatoes, the study found that farmers store tubers for planting in the next season, or to aggregate for collection by traders, but not for aiming for better prices in the future. Most farmers sell immediately after harvest. In Bomet where the variety Dutch Robijn is preferred for processing into chips or crisps, storage was considered a pressing challenge. The study concluded that the main driver for investment in storage was to ensure continuity of product supply for processing, perhaps by rural brokers and was not warranted for smallholder farmers.

There are various preference rankings that farmers, processors, and consumers take into consideration when adopting or choosing a variety. A study by Sinelle (2018) interviewed 289 farmers in six of the major potato production regions in Kenya. The results showed that market demand and income are the most important criteria for choosing a variety, more than pest and disease and adverse weather impacts. Therefore, even if a variety does not have the best resistance to a disease such as Late Blight, if it has a high yield potential and there is demand for it in market, farmers generally preferred to grow it instead of more resistant varieties. This was the same for both women and men farmers.

A recent study using focus group discussions with male and female potato farmers and a household survey in three important potato growing regions in Northwest Kenya revealed that availability and access of healthy seed was a critical factor for varietal choice (Kwambai et al., 2024). The seed sources were mostly from farmer-kept seed. In addition, market demand played a dominant role in variety selection by farmers. Shangi was the predominant variety in the market.

### 2.2 Variety design

Breeding programs are tasked with addressing challenges from various angles, including a crop's production, the successful adoption of the breeding products by end-users, as well as significant market penetration. This entails a process of prioritization of objectives and desired trait combinations to be selected for. Therefore, variety design is the first step in the development of a new variety while incorporating knowledge and learnings from the development of previous varieties in the breeding cycle (e.g., what needs to be improved or changed). Consequently, as promoted and implemented by the CGIAR RTB program and Excellence in Breeding Platform (EiB),^3^ breeders and ideally multi-functional teams first determine the market segments that will be the targets of the breeding program. The market segments are comprised of the basic agronomic, demographic and economic characteristics of the geographic region being targeted. This is complemented with production and consumer components. Thereafter, product profiles are developed that correspond to each market segment. A product profile is comprised of a specific list of key traits with defined levels being targeted in a new product (variety) that will achieve the goals of the breeding program. The concepts of market segments and product profiles are described in Donovan et al. (2022).

### 2.3 Potato breeding scheme at CIP

Breeding of vegetatively propagated crops such as potato comes with unique challenges and these have been described extensively (Jansky and Spooner, 2018; Bradshaw, 2022; Lindqvist-Kreuze et al., 2024). This process usually takes 8–10 years from crossing to variety release. CIP was established in 1971 with an initial mandate on potato improvement for production in developing countries (Lindqvist-Kreuze et al., 2024). It was located in Peru, which is part of the potato's center of origin and diversity. The main traits targeted for selection in the CIP breeding program had to do with agroecological conditions and the main diseases under those conditions. Resilience for low-input production and poor soils, and quality traits were not prioritized. Having good dormancy for around 3 months was taken for granted to allow storage of potato tubers until the next planting season. A study in 2015 in Kenya stated that 3 months storage are needed by farmers to reach periods of higher prices, and 4 months needed by processors to avoid periods of limited supply (Manyasa, 2015).

### 2.4 The need to address gender for social inclusion and equity

By taking into consideration the social dynamics of gender relations at various levels, and gender roles along the value chain, gender research can help identify constraints and challenges faced by women that can affect variety adoption. This is especially relevant in cases where production systems and subsequent post-harvest activities are carried out differently by men and women (Christinck et al., 2017). Variety adoption studies have shown that women producers may be less likely to adopt improved varieties (Polar et al., 2021) for a number of reasons. These can include the social context as well as trait preferences, especially quality traits.

Studies in various crops and livestock have shown certain desirable traits to actually affect the livelihoods of women producers, processors and consumers which resulted in lower adoption of improved varieties and breeds as reviewed in Polar et al. (2021). For instance, in groundnut in Malawi, a variety was developed with resistance to a major disease, but it also had hard shells. Women opted to grow another variety, less resistant but with soft shells, as the hard shells made shelling much more difficult and also increased the risk of contamination with mycotoxins (Tufan et al., 2018). This led to a refocusing of the breeding program, to develop resistant varieties with soft shells. Ease of peeling of cassava was especially highlighted by women in a study in Nigeria and Cameroon as peeling is mostly done by women and children (Ndjouenkeu et al., 2021).

In the potato crop, women play important roles in production, home consumption and marketing. In Uganda, a study of gender-preferred potato traits showed that although there were similar preferences between men and women for sensorial quality traits, women ranked attributes that are easier for processing higher than men, since women are normally more engaged in processing (Mudege et al., 2021). Market preferences, which were gender neutral included appearance of the tuber, namely red skin and yellow flesh. Women preferred large size of tubers and mealiness. From this approach that also included gender specialists, it became apparent that breeders must consider gender roles, social norms, and market preferences in addition to the usual agronomic traits prioritized in breeding programs. Resistance to Late Blight was highlighted by both men and women, as well as cooking qualities. Interestingly, varieties with long dormancy such as KACHPOT 1 were not popular, whereas the variety Victoria was popular due to its short dormancy, early maturity, and large tubers, even though it was not market-preferred and was not considered to have a good taste.

For women, the primary purpose for growing potato in Uganda is one of food security-so their production plots are subsistence farming, with less inputs, and cooking by boiling (Kisakye et al., 2020). Priority traits for boiled potato were appearance, color, size, texture and dry matter content. As the crop becomes more commercialized, the report showed the crop to become more male-dominated. Moreover, women farmers having limited mobility and many domestic responsibilities, are more restricted in participating in marketing of the potatoes. Market demand by traders and consumers was found to be crucial for variety selection by farmers. Traders grading potatoes looked at the variety, appearance and size, maturity level, water content and damage. The report showed NAROPOT4, Victoria and Kinigi as the most popular varieties grown due to their disease resistance and high yields. Interestingly, the report also noted that Shangi is becoming popular in eastern Uganda, though they did not mention its short dormancy trait as a reason. Organized storage is also a problem in Uganda and most likely across potato growing areas in East Africa, leading to high post-harvest losses. It will be interesting to see how this affects the expansion of Shangi and the development of new product profiles for Uganda.

### 2.5 GBI approach to define customer segments and gender-sensitive traits

Breeding objectives historically have mainly sought to improve yields and biotic and abiotic resistance. Due to issues related to low adoption of improved varieties stemming from lack of attention or strategies to address needs and preferences of stakeholders along value chains, the CGIAR established the Gender in Breeding Initiative (GBI)^4^ in 2016, led by the CGIAR Research Program on Roots, Tubers & Bananas (RTB). The aim was to mainstream gender into breeding programs, especially to enhance the design of new varieties. The objective was to develop a common ground and facilitate communication and collaboration between breeders and gender specialists (Polar et al., 2022). The initiative established procedures to create multidisciplinary teams. These were comprised of breeders, social scientists, gender specialists, food scientists and others to jointly design varieties for breeding programs by defining and perfecting product profiles. Moreover, the initiative developed tools (the so-called G+ Tools) to describe and define gendered customer segments and a gender-responsive product profile which are now available as manuals (Ashby and Polar, 2021a,b). This has led to various CGIAR and partner organization breeding programs to adopt the G+ tools and include gender specialists in their breeding teams (Polar, 2019).

## 3 Analysis

The evolution of the Target Product Profiles (TPPs) went through an iterative process, through the involvement of the GBI and active participation of the breeders. What started with breeders only, evolved into product design teams that involved experts from multiple disciplines. This resulted in the definition of TPPs that responded to the needs and preferences of specific and relevant market segments.

### 3.1 Early product profiles

Early attempts by CGIAR breeding teams to define product profiles, ranked traits as either “must have” or “good to have”. This was followed by a quantified description of the desired level of the trait (such as maximize, reach specified level, maintain a certain minimum level, etc.), and a unique rank or priority was assigned. A target trait level was determined, defined in terms of the levels of the predominant variety for that region to be replaced. The traits were then ranked taking into consideration factors such as the genetic variability available for that trait, the heritability of the trait, the ease of measuring the trait, in addition to the expected impacts of the trait.

Following these criteria, a draft potato product profile was developed for the tropical highlands of Africa and Latin America in 2017 by the CIP potato breeding program (see Table 1, T. Mendes, personal communication). At this time, the market segment was quite broad and did not differentiate between fresh market and processing types. Moreover, most potato processing is through cottage industries that do not utilize processing type potatoes. Instead, they use dual-purpose types to deal with under-developed markets for processed potatoes such as chips and crisps. In this product profile, table quality traits were rather vague, and dealt more with the issue of rises in glycoalkaloid content under hot conditions (Gastelo et al., 2014). Dormancy was mentioned but given at least 60 days for two cropping seasons a year, or 90 days for one season per year, with the greatest emphasis in the product profile being on resistance to various major diseases and nematodes. Nevertheless, this initiated a process of quantifying and ranking traits to be included in a breeding program, and gathering feedback from breeders and other stakeholders to design the product profile.

**Table 1:** Draft potato product profile for tropical highlands.

**Region/market segment**	**Trait (economic, sustainability, livelihood) and value**	**Target trait level**	**Market priority**	**Selection objective**
**African and Andean highland tropics**				
Fresh market and processing	Yield	10% greater than X variety across a range of soil and management conditions	*1*	Maximize
	Table quality	tuber appearance and cooking type (check X), glycoalkaloid concentration < 15 ppm.	1	Reach threshold
	Earliness	< 110 days maturity	1	Reach threshold
	Resistance to late blight	Late blight susceptibility scores < 3	1	Reach threshold
	Resistance to PVY	Extreme resistance to PVY	1	Reach threshold
	High Fe or Zn concentration	At least 35 ppm Fe or 30 ppm Zn	3	Opportunistic
	Drought tolerances (water productivity)	TBD (ratio of fresh tuber yields to applied water expressed as kg ha1 mm)	3	Reach threshold
	Good storability/dormancy	Unimodal >90 days and Bimodal to >60 days—sprouting with low water loss in storage	1	Reach threshold
	PLRV resistance	Resistance to PLRV as high or higher than variety X.	3	Opportunistic
	PVX resistance	Extreme resistance to PVX	3	Opportunistic
	Chipping ability	Chip score < 3	1	Reach threshold
	PCN resistance	No symptoms of PCN in inoculated plants and tubers	3	Opportunistic
	Bacterial wilt resistance	No symptoms in inoculated plants and tubers	3	Opportunistic

The product profiles were further refined in a workshop bringing together breeders, gender specialists, food scientists and economists in 2020 (Friedmann et al., 2020). First, the market segments were determined according to a framework provided by the Excellence in Breeding Platform. Then specific traits for each category were listed in the associated product profile following quantity traits and quality traits, each with defined scales and minimum scores required. The workshop further helped define the market segment, that was too broad for the draft potato product profile. Discussions also centered on how to access the needed data to properly establish the market segment and the need for social scientists and economists to work with breeders to develop it. It also became apparent that a crop usage category needed to be added to the market segment template. This would help to better elucidate the quality traits to be considered in the product profile. It also brought to light the value of the G+ tools mentioned above, and the need to find better strategies to incorporate the tools into the variety design process. This was a good exercise to bridge to what later became more rigorous establishment of market segments and target product profiles (TPPs) with further inputs from the EiB and the establishment of the CGIAR Market Intelligence Initiative (MIPPS).^5^

In late 2018, the CIP potato breeder based in Kenya participated in a GBI workshop to fine tune the G+ tools, test them with real-world examples of breeding programs, and provide feedback and how to best incorporate and implement them in breeding programs (Hershey, 2018). The workshop looked at the potato breeding program in Kenya as one of the case studies to apply the G+ tools. For the customer profile tool, the case study segmented the customers based on geography- concentrating on tropical highlands, and then disaggregating based on population size, gender and age in the major potato producing counties. However, as Shangi was already estimated to be grown in 80% of the production area, it was not possible to do such a segmentation according to these criteria. More research on gender roles was suggested, on the use of the potato product, and what the end markets are, using gender disaggregated data, as well as information on age, income, and separating consumers as rural or urban in order to re-assess to develop a customer segmentation that might support gender equality and inform the development of the product profiles. There were many points raised in the feedback sessions. Among them, workshop participants suggested that analyzing what traits are important in varieties that are currently grown can inform the development of effective product profiles for new varieties. This led to looking more closely at the traits of Shangi. The agronomic and quality characteristics of the variety are good, but not outstanding. The main trait that stood out was its short dormancy. This was counter-intuitive, given the importance of a mid-to long dormancy for effective storage of potato seed tubers, and that breeding programs usually selected for storability (longer dormancies) historically. This led to the breeding team to reconsider its assumptions regarding short dormancy and triggered their interest and curiosity about why and how this trait affected potato variety preferences.

A follow up workshop in Nairobi in 2019 aimed to share various experiences using a number of tools and strategies to integrate social differentiation and gender into product profile development (Polar, 2019). The group on potato evaluated survey data taken from 120 farmers (50% women), 22 processors and restaurant owners, 12 traders (mostly men), and 40 retailers (mostly women) from Uganda. Sensorial and organoleptic data were found to be still missing. It was determined that the breeding program still needed to properly define quality traits, translate farmer preferences to standardized scales for use by breeders, establish cross-functional teams, increase interaction with processing industry and improve communication with NARS and government stakeholders.

### 3.2 Market intelligence for East Africa market segment

Although much work has been done to elucidate varietal preferences of farmers through participatory variety selection (PVS), more systematic, accurate, forward-looking and scalable approaches are needed to capture the size and nature of current and future demand for varieties. Therefore, the CGIAR Initiative on Market Intelligence (MIPPS) is striving to standardize and develop tools for breeding teams to define market segments that will inform the design of TPPs for each segment. In this manner, information and data are collected to prioritize and align investments in breeding pipelines and seed systems (Donovan et al., 2022).

For potato, consumer requirements have a strong weight in determining the market segments. The data collected has been compiled in a Seed Product Market Segment Database^6^ including potato, with currently nine market segments. For example, the market segment for table potato in the highlands of East Africa covers a target area of 412,000 hectares.^7^ A dashboard provides the main criteria defining the market segment. Data characterizing the population in the region such as the population living in poverty and being malnourished can inform on the potential impact of a breeding pipeline investment. This segmentation provides the basis for developing the TPP for this segment. It is noteworthy that early maturity, and short dormancy are highlighted for this market segment.

### 3.3 G+ tools to inform potato market segments and target product profiles for East Africa

The development of the information on potato for the dashboard mentioned above was extensively described by Ojwang et al. (2023). In it, the authors analyzed the International Potato Center (CIP) and partners' potato breeding programs' potential impacts according to indicators of poverty, malnutrition, and gender. Using the seed product market segmentation blueprint developed by the EiB described above, the study identified and estimated the sizes of the market segments at subregional levels. A qualitative analysis described the sub-regions considering target populations of environments (TPEs) which are comprised of sets of farmers and seasons where a variety will be grown. The production systems were then described (e.g., rainfed vs. irrigated), as well as input systems and maturity. The criteria for consumer preferences then captured parameters such as cooking time, nutritional enhancement, flesh color, mealiness and hardness and the use of the product such as fresh market, consumption at home, or processing. This resulted in nine market segments being described. The market segments were then characterized quantitatively based on estimated size and opportunities for poverty alleviation, nutrition and gender equity outcomes using data from open-access databases.

The East Africa region had two market segments defined by use, household consumption (termed “table potato”) and dual purpose (suitable for both commercial processing and household consumption). Fast cooking, early maturity and short dormancy were determined as the defining traits for this sub-region. The study then went further to estimate the potential poverty and nutrition impacts of investments in the respective market segments. The analysis showed more stunted children are found in the table potato market segments than the dual-purpose segment. The East Africa potato pipeline was shown to have 10.7 million stunted children in the table segment and 2 million in the dual-purpose segment. There was a 22% prevalence of undernourishment (Ojwang et al., 2023).

Using multidisciplinary teams, the G+ tools were then used to evaluate the gender-responsiveness of the breeding programs. The G+ Customer Profile Tool was used to map the customers for various products in the different market segments using gender-disaggregated data (Ojwang et al., 2023). The G+ Product Profile Tool was used to examine potential harmful as well as beneficial effects of specific traits in the product profiles. The teams looked at drudgery and time poverty, control over critical on-farm resources, access to inputs and control over benefits such as income from sales of the potato crop. The results identified traits associated with the “do no harm” concept, receiving a score to “amend”. Therefore, the study showed that future gender-responsive breeding programs should take account of gendered quality traits such as taste, that are currently missing in the product profiles. The “do no harm” analysis highlighted the need to address gender relations to mitigate unequal benefit sharing. This kind of market segment analysis allows the breeding program to evaluate its breeding pipeline, looking at investments and potential impacts in the various segments across and within countries.

A recent study looked at end user preferences to inform product profiles in potato breeding in the Rakai and Kabale districts in Western Uganda (Nantongo et al., 2023). The G+ tools were used to evaluate priority quality traits for acceptance and adoption. Physico-chemical methods including instrument-based texture measurements such as penetration force and near-infrared spectroscopy (NIRS) were used to evaluate quality traits, so that breeders could use these in selecting material. The study followed a five-stage stepwise process to evaluate the quality characteristics for boiled potato as described in Forsythe et al. (2021). From a gender perspective, large tuber size, fast cooking time, moderately firm and good taste were identified as essential traits, as women are mainly involved in cooking the potatoes. For example, large tubers resulted in less waste due to peeling. Shangi was classified as soft and less mealy.

Another study applied the G+ tools using a multi-functional team of value chain actors to evaluate and modify a TPP developed under the Partnership for Seed Technology Transfer in Africa (PASTTA) project for table potato in Kenya, using Shangi as the benchmark (Mwende Mutiso et al., 2024). The profile targeted six traits: disease and pest resistance, tuber yield, earliness, dry matter content and shelf-life. However, dormancy was not highlighted among the key traits as most varieties have dormancy periods of a few months. Nevertheless, in analyzing the key traits through the gender lens, issues were identified that could bring forth the importance of short dormancy. Male farmers preferred earliness that allowed them to grow the crop thrice a year thus increasing profits because early potatoes are sold at a higher price when there is no glut. This is possible with varieties with short dormancy such as Shangi. Shelf-life was more controversial, as long shelf-life allows for storing potatoes with reduced post-harvest losses and getting higher prices after the harvest glut season. However, women retailers could be disadvantaged as they usually have less access to storage facilities than male counterparts. In addition, in respect to tuber seed availability, longer shelf-life that is negatively correlated with short dormancy, would prevent women from planting their own saved seed, especially when planting two seasons a year (Mwende Mutiso et al., 2024).

### 3.4 Shangi variety traits and development of the new potato product profile for Kenya

Dormancy is a physiological state in potato tubers, that affects production and storage. If the tuber is still dormant when planted, it will not start sprouting properly, thus affecting yields. If stored when not dormant, it will sprout prematurely and lead to spoilage and losses, whereas a dormant tuber will store stably until it sprouts (Kwambai et al., 2023). For production in tropical highlands, when more than one crop cycle is grown per year, a short dormancy of 1 or 2 months is required. In temperate regions with long winters, long dormancies for storage of both seed and ware potatoes are required.^8^

A study was carried out to examine the dormancy of 47 different varieties in Kenya grown at three altitudes over two seasons (Kwambai et al., 2023). As in Kenya potatoes are grown in the long rainy season as well as the short rainy season at mid- to high altitudes, dormancy was evaluated and compared between the seasons. Ideally, breeders should select adapted varieties with a dormancy profile that can balance the ware storage with the optimal seed physiological age for planting, tailored for specific growing conditions (season and altitude). Shangi, Dutch Robijn, and Tigoni were the local checks in the study. There were large differences between the genotypes on days to dormancy release, with Shangi being the shortest with an average of 53.8 days to sprout. Other popular varieties had much longer dormancies such as 75.7 for Dutch Robjin and 72.3 for Asante.

As Shangi became a prevalent variety in most potato growing regions, preferred both by farmers, traders, and processors, it became necessary to re-evaluate commonly held assumptions in regard to storability and dormancy traits. This became apparent when the potato breeders, together with other stakeholders, economists, food technologists, gender specialists, went through the process of looking at variety design following the principles and strategies of demand-led breeding and the GBI. Consequently, market segments were designed looking at the whole potato value chain, both for fresh table produce, and processing into chips or crisps. Moreover, by examining the customer segments looking at gender but also social contexts of smallholder and disadvantaged farmers such as access to markets, inputs, labor, etc. it became apparent that availability at the right time of potatoes for consumption, processing or planting was of prime importance. Especially in the context of lack of storability infrastructure and poor access to distant markets due to poor infrastructure and undeveloped market structures (Manyasa, 2015). Shangi's very short dormancy, which had been assumed to be negative trait both for farmers, women processors and consumers, and cottage processors, was actually meeting the needs for available product and planting material given the storage and market constraints (Manyasa, 2015; Mwende Mutiso et al., 2024). In this manner, a need for potatoes could be met year-round. Moreover, some farmers, due to the short dormancy, were able to shift production to three seasons a year.

The iterative process described in this section led to the formulation of two updated TPPs for table potato and for dual-purpose (table and processed) potato for East Africa as well as another seven TPPs for other CIP potato breeding team target market segments (see text footnote 7). The TPP for table potato for East Africa is shown in Table 2. The TPPs follow the EiB structure for defined market segments followed by the traits, their description, desired levels, and a ranking of importance. By evaluating the market demand for Shangi and examining what traits contribute to its popularity, the dormancy trait is now part of the TPP. The profile now requires the new variety to be bred to have a short dormancy of under 60 days for production areas where two seasons are produced in 1 year (Bimodal). For the processing potato TPP, this is not a requirement, as part of the crop will need to be stored to ensure continuous supply to the processors. In addition, the TPPs have defined essential and nice to have tuber quality traits related to flavor, texture, cooking quality and cooking time, in response to the various studies that showed tuber taste and cooking attributes to be important for women (Mudege et al., 2021; Nantongo et al., 2023).

**Table 2:** Updated target product profile for table potato^a^ for East Africa.

**Trait type**	Trait name^*^	**Scale option**	**Trait requirement**	**Desired score**
Agronomic	Tuber yield	Tons/ha	Nice to have	10% above check
	Marketable tuber yield	Tons/ha	Essential: improve	10% above check
Biotic—disease	Late Blight susceptibility	1 to 9	Essential: improve	< 3
	Potato Virus Y resistance	1 to 7	Essential: threshold	1
	Potato Virus X resistance	1 to 7	Nice to have	1
	Bacterial Wilt resistance	1 to 6	Nice to have	< 3
Biotic—pests	Potato Cyst Nematodes resistance	1 to 9	Nice to have	< 3
Quality—analytical	Tuber dry matter content	%	Nice to have	18–20
	Chips oil absorption rate	%	Nice to have	< 2
	Tuber flavor	1 to 5	Essential: threshold	>4
	Tuber cooking quality	1 to 7	Nice to have	< 5
	Tuber cooking time	min	Essential: threshold	< 10
	Tuber glycoalkaloids concentration	ppm	Essential: threshold	< 15
	Tuber dormancy period	days	Essential: threshold	< 60
Quality—visual	Predominant tuber skin color	1–9	Essential: threshold	6, 5 or 1
	Predominant tuber flesh color	1–8	Essential: threshold	4, 2 or 1
	Chips color	1–5	Nice to have	< 2
	French fries color	1–5	Nice to have	< 2
	Tuber depth eye	1–9	Essential: threshold	< 3
	Tuber texture	1–5	Essential: threshold	>4
	Tuber appearance	1–9	Essential: threshold	>5
	Tuber shape	1–8	Essential: threshold	2–7
	Tuber uniformity	1–9	Essential: threshold	>5

^*^Based on the potato ontology available at https://cropontology.org/term/CO_330:ROOT.

^a^https://glomip.cgiar.org/target-product-profiles.

## 4 Conclusions

The ongoing efforts by CGIAR and partner breeding teams to shift to more demand-led breeding while establishing processes for more standardized variety design and genetic material advancement decisions using multidisciplinary teams is leading to well-characterized breeding pipelines based on priority market segments.^9^ For the potato breeding program, this has resulted in nine TPPs linked to nine market segments, each with ranked sets of traits with desired ranges. Not only does this make more efficient use of limited resources, but it allows the monitoring and evaluation of breeding programs as well as capturing valuable learnings of processes and what works and what needs improvement. The expansion of this strategy to gender-responsive breeding using the tools and approaches of the GBI is enhancing the relevance and future adoption of new improved varieties coming thru the breeding program pipelines.

In the case of potato in East Africa, gender considerations have elevated the priority of quality traits such as taste (Mudege et al., 2021; Ojwang et al., 2023) and the need to develop effective assays for screening such traits (Nantongo et al., 2023). Even though short dormancy was found to be gender-neutral, the process of analyzing the product profiles through a gender lens, using the G+ tools made the breeders and multi-functional teams aware of the importance of re-evaluating long held conceptions about prioritizing long dormancy in all breeding contexts. The gender analysis of long shelf-life with conflicting views of benefit and harm to women raised the issue of how to handle the interaction with dormancy (Mwende Mutiso et al., 2024). In East Africa, where two and sometimes three potato crops are grown in the year, together with the lack of storage facilities (needed for long dormancy varieties), the dominant popularity of the Shangi variety is apparent. Shangi provides much needed ware and seed availability at all times. Therefore, the above process resulted in a dramatic change in the corresponding TPP, prioritizing short dormancy instead of having long dormancy as a given in all the breeding material. Therefore, attention to gender triggers closer attention to different segments of the population and can help breeding programs be more inclusive and responsive to a diversity of needs.

As mentioned in Mwende Mutiso et al. (2024), evaluating traits in a TPP using the “do no harm analysis” provides insights into possible impacts on gender equality. This is especially important in relation to commercialization of the crop, where men dominate and women may not share in the benefits, even if they must provide more labor for the commercial crop. For positive traits such as higher yields, these kinds of considerations must be taken into account, and the programs with improved higher-yielding varieties may need to accompany their release with strategies that mitigate such negative impacts on women. For example, early maturing varieties can be accompanied by extension activities to promote staggered planting and piecemeal harvesting to mitigate the burden of labor for women that have many other chores during planting and harvesting (Mwende Mutiso et al., 2024). In addition, in considering traits with a positive impact, this can bring added benefits to a new released variety, enhancing productivity, food security, and community resilience.

The changes to breeding program priorities to address the need for short dormancy reveals the farmers', processors' and traders' intention to have better control over their access to seed and product. Thus, they take advantage of this trait to address their limitations stemming from lack of storage facilities and underdeveloped market structures limiting access to distant markets.

## Data Availability

Publicly available datasets were analyzed in this study. This data can be found here: https://glomip.cgiar.org.

## Footnotes

^1^National Potato Council of Kenya 2023- https://npck.org/.

^2^NPCK Kenya Variety catalog-2021- https://npck.org/catalogue/.

^3^ https://excellenceinbreeding.org/

^4^ https://gender-portal.rtb.cgiar.org/breeding/

^5^ https://www.cgiar.org/initiative/market-intelligence/

^6^ https://ebs.excellenceinbreeding.org/wp-content/uploads/2023/03/MS-public.html

^7^ https://glomip.cgiar.org/target-product-profiles

^8^ https://blog.potatoworld.eu/dormancy-and-sprouting

^9^ https://ebs.excellenceinbreeding.org/wp-content/uploads/2023/03/MS-public.html
